# Supplementary material for: Transport of Non-Steroidal Anti-Inflammatory Drugs across an Oral Mucosa Epithelium In Vitro Model
Source: Pharmaceutics. 2024 Apr 15;16(4):543. doi: 10.3390/pharmaceutics16040543 (PMC11054638; doi:10.3390/pharmaceutics16040543)
Supplement: Supplementary file 1 [file pharmaceutics-16-00543-s001.zip › pharmaceutics-2937427-supplementary.pdf]

Table S1. Overview of PS values (blank, all: cell + blank, cell) and permeability values of cell (PC) and cell + blank (PE) from transport studies with NSAIDs, diazepam and carboxyfluorescein (CF) with and without inhibitor (verapamil, probenecid) as well as measured TEER values at beginning of experiments. Results shown as mean ± SD from three independent experiments (N=4-8). Significances compared to PC or PE values of diazepam values per experiments.

			PS blank [µL/min]	PS all [µL/min]	PS cell [µL/min]	PC [µm/min]	PE [µm/min]	TEER [Ω*cm^2]
Celexocib	w/o Inhibitor	Diazepam	0.18 ± 0.003	0.11 ± 0.02	0.38 ± 0.19	11.44 ± 5.76	3.40 ± 0.70	201.35 ± 42.35
		CF	0.89 ± 0.08	0.13 ± 0.03	0.15 ± 0.04	4.38 ± 1.12*	3.74 ± 0.85	
		Celexocib	0.06 ± 0.03	0.01 ± 0.005	0.01 ± 0.006	0.44 ± 0.19**	0.36 ± 0.15***	
	Verapamil	Diazepam	0.13 ± 0.05	0.09 ± 0.05	0.41 ± 0.35	12.29 ± 10.31	2.82 ± 1.46	185.56 ± 24.59
		CF	0.77 ± 0.06	0.14 ± 0.03	0.18 ± 0.04	5.33 ± 1.19*	4.31 ± 0.83**	
		Celexocib	0.09 ± 0.03	0.03 ± 0.03	0.07 ± 0.08	2.1 ± 2.24**	1 .00 ± 0.77**	
	Probenecid	Diazepam	0.20 ± 0.004	0.14 ± 0.004	0.46 ± 0.04	13.74 ± 1.17	4.08 ± 0.11	243.18 ± 88.75
		CF	0.97 ± 0.04	0.14 ± 0.03	0.16 ± 0.05	4.76 ± 1.36*	4.06 ± 1.02	
		Celexocib	0.10 ± 0.05	0.05 ± 0.04	0.33 ± 0.36	6.05 ± 6.014*	1.63 ± 1.13***	
Diclofenac	w/o Inhibitor	Diazepam	0.17 ± 0.01	0.11 ± 0.03	0.44 ± 0.35	13.01 ± 10.37	3.14 ± 0.95	246.79 ± 79.55
		CF	0.87 ± 0.17	0.14 ± 0.02	0.16 ± 0.04	4.89 ± 1.12**	4.05 ± 0.73	
		Diclofenac	0.18 ± 0.02	0.07 ± 0.01	0.12 ± 0.03	3.64 ± 0.84**	2.11 ± 0.22	
	Verapamil	Diazepam	0.13 ± 0.03	0.1 ± 0.03	0.68 ± 0.69	7.56 ± 1.51	2.87 ± 0.91	178 ± 55.18
		CF	0.85 ± 0.19	0.12 ± 0.02	0.14 ± 0.03	4.16 ± 0.81	3.54 ± 0.63*	
		Diclofenac	0.17 ± 0.01	0.07 ± 0.01	0.12 ± 0.03	3.54 ± 0.94	2.06 ± 0.3	
	Probenecid	Diazepam	0.15 ± 0.03	0.12 ± 0.01	0.57 ± 0.21	16.88 ± 6.19	3.47 ± 0.3	218.4 ± 52.1
		CF	0.98 ± 0.48	0.15 ± 0.03	0.19 ± 0.05	5.65 ± 1.51***	4.59 ± 1.04	
		Diclofenac	0.2 ± 0.04	0.1 ± 0.05	0.22 ± 0.15	6.66 ± 4.59***	2.98 ± 1.37	
Ibuprofen	w/o Inhibitor	Diazepam	0.21 ± 0.03	0.12 ± 0.02	0.31 ± 0.08	9.23 ± 2.34	3.65 ± 0.64	202.78 ± 47.09
		CF	0.98 ± 0.2	0.14 ± 0.05	0.17 ± 0.07	4.98 ± 2.03*	4.16 ± 1.44	
		Ibuprofen	0.21 ± 0.03	0.1 ± 0.01	0.19 ± 0.05	5.77 ± 1.48*	2.94 ± 0.3	
	Verapamil	Diazepam	0.12 ± 0.06	0.08 ± 0.02	0.39 ± 0.35	7.22 ± 0.81	2.45 ± 0.68	174.94 ± 33.42
		CF	0.93 ± 0.14	0.14 ± 0.04	0.17 ± 0.07	5.14 ± 1.98	4.21 ± 1.33*	
		Ibuprofen	0.21 ± 0.02	0.11 ± 0.01	0.24 ± 0.04	7.13 ± 1.24	3.27 ± 0.33	
	Probenecid	Diazepam	0.14 ± 0.01	0.09 ± 0.02	0.29 ± 0.16	8.65 ± 4.62	2.74 ± 0.54	171.92 ± 44.76
		CF	1.06 ± 0.29	0.17 ± 0.05	0.21 ± 0.09	6.15 ± 2.56	4.95 ± 1.46*	
		Ibuprofen	0.22 ± 0.02	0.12 ± 0.03	0.26 ± 0.11	7.87 ± 3.24	3.47 ± 0.76	
Piroxicam	w/o Inhibitor	Diazepam	0.19 ± 0.02	0.12 ± 0.04	0.45 ± 0.24	13.4 ± 7.01	3.71 ± 1.07	183.01 ± 56.55
		CF	0.91 ± 0.06	0.13 ± 0.02	0.15 ± 0.02	4.38 ± 0.62***	3.77 ± 0.46	
		Piroxicam	0.18 ± 0.02	0.08 ± 0.03	0.16 ± 0.08	4.86 ± 2.41***	2.44 ± 0.76**	
	Verapamil	Diazepam	0.09 ± 0.02	0.05 ± 0.03	0.24 ± 0.36	2.97 ± 2.32	1.62 ± 0.94	167.74 ± 24.09
		CF	0.85 ± 0.1	0.13 ± 0.01	0.15 ± 0.01	4.39 ± 0.31	3.73 ± 0.2***	
		Piroxicam	0.22 ± 0.03	0.1 ± 0.03	0.2 ± 0.07	6.00 ± 1.99*	3.07 ± 0.79***	
	Probenecid	Diazepam	0.12 ± 0.03	0.1 ± 0.01	0.32 ± 0.14	9.63 ± 4.3	2.93 ± 0.26	176.59 ± 52.18
		CF	0.91 ± 0.09	0.15 ± 0.02	0.18 ± 0.04	5.39 ± 1.13	4.46 ± 0.74**	
		Piroxicam	0.18 ± 0.02	0.09 ± 0.02	0.2 ± 0.06	5.81 ± 1.73	2.71 ± 0.47	

High-throughput (96.96) qPCR chip

Preparation and set-up was described previously in Lin et al. (2020) (1). In short, RNA samples collected at the 48-hour timepoint of the inflammation studies and generated by pooling two cell-grown inserts were tested as duplicates on the expression of 95 markers and 1 negative control (H_2O). The mRNA samples were amplified with three different primer pools containing 54 primers (PPIA, ACTB, GAPDH, B2M, CLDN1, CLDN2, CLDN3, CLDN4, CLDN5, CLDN6, CLDN7, CLDN8, CLDN9, CLDN10tva, CLDN10tvb, CLDN11, CLDN12tv1, CLDN12tv2, CLDN12tv3, CLDN14, CLDN15, CLDN16, CLDN17, CLDN18tv2a, Cldn19tv1, CLDN20, CLDN21, CLDN22, CLDN24, JAM1, JAM2, JAM3, OCLN, MARVELD2, ZO-1, ZO-2, ZO-3, SLC2A1, SLC7A1, SLC7A5, SLC16A1, ABCB1, ABCC1, ABCC2, ABCC3, ABCC4, ABCC5, ABCG2, INSR, LRP1, TFRC, VEGF-A, CDH5, VWF), 32 primers (CK1, CK4, CK5, CK8, CK10, CK13tv1, CK14, CK16, CK18, CK19, LOR, CDH1, CTNNB1, VIM, MUC1A, MUC1B, MUC3A, MUC5AC, MUC5B, MUC13, MUC15TV2, MUC16, MUC18, MUC20, MUC21, AQP1, AQP3, AQP5, AQP7, AQP9, AQP10, AQP11) or 9 primers (CLDN25tv1-4, CLDN25tv7, CLDN26, CLDN27, MFSD2A, K7, DSG3, AMYα1B, PECAM1). The nomenclature of claudin follows Mineta et al. (2011) (2). Quality control of generated data excluded Ct measured outside of the marker's melting temperature ± 1.5 °C. To calculate the relative expression for each marker, the relative quantitative value was first calculated by subtracting the mean Ct values of control samples from the Ct values of treated samples and potentiating the resulting ΔCt by the power of 2 (=$2^{\Delta Ct}$). The geometric mean of housekeeping genes (ACTB, GPDH, B2M; PPIA was excluded due to high variances) was calculated for each sample. Next, the relative quantitative value of each marker was divided by the calculated geometric mean of housekeeping genes of the respective sample. Subsequent expression values of treated samples were normalised to expression values of control samples to give n-fold expression, shown in Table S2.

Table S2. Overview of mRNA expression after inflammation studies analysed with a high-throughput (96.96) qPCR chip. Tested RNA samples were collected after three independent experiments with 4 treatment groups (Control, INF, INF+Ibu, Ibu) per experiment (N=12). Statistical analysis was performed as two-way ANOVA with post hoc Holm–Sidak test with *$p<0.05$, **$p<0.01$, ***$p<0.001$ and α=0.05. No expressions in all samples of 4 groups were seen for CLDN19tv1, CLDN26, AQP7, CTNNB1, CDH5, MUC5B, MUC13 and MUC16.

	**Control**		**INF**		**INF+Ibu**		**Ibu**	
**Marker**	**mean ± SD**	**N**	**mean ± SD**	**N**	**mean ± SD**	**N**	**mean ± SD**	**N**
CLDN1	1± 0.2	3/3	0.54± 0.05	3/3	0.6± 0.48	3/3	0.52± 0.71	3/3
CLDN2	1± 0.01	2/3	2.63± 1.52	3/3	1.98± 1.39	3/3	5.02	1/3
CLDN3	1± 0.41	3/3	1.31± 0.92	3/3	1.05± 1.14	3/3	1.95	1/3
CLDN4	1± 0.23	3/3	0.2± 0.1	3/3	0.35± 0.29	3/3	0.74± 1.1	3/3
CLDN5	1± 0.49	3/3	2.99± 2.81	3/3	5.34± 4.14	3/3	7.17± 10.76	3/3
CLDN6	1± 0.48	3/3	2.72± 1.27	3/3	3.57± 1.83	3/3	1.83± 0.78	3/3
CLDN7	1± 0.25	3/3	0.87± 0.11	3/3	0.77± 0.6	3/3	0.51± 0.72	3/3
CLDN8	1± 0.27	3/3	0.79± 0.83	3/3	1.24± 1.26	3/3	0.49± 0.46	3/3
CLDN9	1± 0.44	2/3	0.96± 0.41	2/3	1.06± 1.09	3/3	1.07± 1.46	2/3
CLDN10tva	1± 0.39	3/3	*Control<br>8.84± 10.73	3/3	***Control, Ibu<br>14.19± 14.45	3/3	1.49± 2.21	3/3
CLDN10tvb	1	1/3	5.18± 6.41	3/3	8.73± 6.94	2/3	n/a	0/3
CLDN11	1± 0.28	3/3	0.58± 0.02	3/3	0.47± 0.19	3/3	0.6± 0.1	3/3
CLDN12tv1	1± 0.62	3/3	2.74± 0.54	3/3	2.27± 0.67	3/3	0.73± 0.3	3/3
CLDN12tv2	1± 0.31	3/3	0.88± 0.21	3/3	0.79± 0.35	3/3	0.47± 0.35	3/3
CLDN12tv3	1± 0.34	3/3	0.86± 0.17	3/3	0.79± 0.17	3/3	0.79± 0.25	3/3

	Control		INF		INF+Ibu		Ibu	
**Marker**	**mean ± SD**	**N**	**mean ± SD**	**N**	**mean ± SD**	**N**	**mean ± SD**	**N**
CLDN14	1± 0.27	3/3	2.57± 0.9	3/3	4.51± 1.11	2/3	1.51	1/3
CLDN15	1± 0.16	3/3	0.76± 0.09	3/3	0.75± 0.41	3/3	0.98± 0.96	3/3
CLDN16	1± 0.22	3/3	0.3± 0.16	3/3	0.42± 0.17	3/3	1.79± 1.1	3/3
CLDN17	1± 0.35	3/3	0.56± 0.54	3/3	0.87± 0.76	3/3	1.66± 1.15	3/3
CLDN18tv2a	1± 0.09	3/3	3.74± 3.76	3/3	6.78± 5.81 **Control	3/3	6.08± 8.62 *Control	3/3
CLDN20	1	1/3	n/a	0/3	2.35	1/3	n/a	0/3
CLDN21	1± 0.86	2/3	16.28± 21.78	3/3	23.81± 25.7	3/3	1.29	1/3
CLDN22	1± 0.28	2/3	1.68± 0.73	3/3	1.7± 1.42	3/3	0.4± 0.53	3/3
CLDN24	1± 0.77	3/3	9.78± 5.33	3/3	4.2± 3.5	3/3	0.62	1/3
CLDN25tv1-4	1± 0.23	3/3	0.67± 0.34	3/3	0.69± 0.44	3/3	0.65± 0.77	3/3
CLDN25tv7	1± 0.73	3/3	1.76± 0.59	3/3	1.44± 1.1	3/3	1.34± 0.38	2/3
CLDN27	1± 0.23	3/3	5.05± 5.07	3/3	12.04± 14.69	3/3	0.26	1/3
JAM1	1± 0.1	3/3	0.59± 0.06	3/3	0.5± 0.36	3/3	0.5± 0.67	3/3
JAM2	1	1/3	1.34± 0.7	3/3	2.71± 0.05 *Ibu	2/3	0.55	1/3
JAM3	1	1/3	0.85± 0.5	3/3	0.73	1/3	n/a	0/3
ZO-1	1± 0.2	3/3	0.98± 0.09	3/3	0.93± 0.65	3/3	0.91± 0.81	2/3
ZO-2	1± 0.24	3/3	1± 0.34	3/3	0.79± 0.28	3/3	1.26± 0.5	3/3
ZO-3	1± 0.09	3/3	0.07± 0.01	3/3	0.03± 0.02	3/3	0.51± 0.84	3/3
MARVELD2	1± 0.21	3/3	0.67± 0.2	3/3	1.15± 0.35	3/3	2.92± 1.97	3/3
OCLN	1± 0.31	3/3	0.25± 0.08	3/3	0.99± 0.79	3/3	1.16± 1.05	2/3
ABCB1	1	1/3	1.04± 0.67	3/3	1.42± 0.62	3/3	1.01± 0.04	2/3
ABCC1	1± 0.08	3/3	1.16± 0.24	3/3	1.13± 0.23	3/3	1.26± 0.46	3/3
ABCC2	1± 0.42	3/3	1.51± 0.48	3/3	0.88± 0.47	3/3	0.7± 0.65	3/3
ABCC3	1± 0.45	3/3	1.16± 0.20	3/3	1.35± 0.74	3/3	0.97± 1.21	3/3
ABCC4	1± 0.22	3/3	6.57± 1.77 ***Control, INF+Ibu, Ibu	3/3	1.55± 0.87	3/3	1.46± 0.5	2/3
ABCC5	1± 0.1	3/3	0.32± 0.05	3/3	0.37± 0.23	3/3	0.69± 0.34	3/3
ABCG2	1± 0.49	3/3	0.44± 0.39	3/3	0.11± 0.06	3/3	0.87± 1.23	2/3
SLC2A1	1± 0.29	3/3	0.22± 0.05	3/3	0.16± 0.06	3/3	0.4± 0.26	3/3
SLC7A1	1± 0.12	3/3	0.52± 0.04	3/3	0.61± 0.21	3/3	0.54± 0.49	3/3
SLC7A5	1± 0.11	3/3	1.02± 0.23	3/3	0.67± 0.55	3/3	0.56± 0.91	3/3
SLC16A1	1± 0.26	3/3	0.83± 0.11	3/3	0.59± 0.48	3/3	0.32± 0.48	3/3
MFSD2A	1± 0.25	3/3	1.3± 0.57	3/3	0.86± 0.58	3/3	0.85± 0.6	3/3
INSR	1± 0.29	3/3	0.57± 0.13	3/3	0.42± 0.24	3/3	0.52± 0.39	3/3
LRP1	1± 0.17	3/3	0.4± 0.15	3/3	0.21± 0.09	3/3	0.68± 0.36	3/3
TFRC	1± 0.14	3/3	1.18± 0.21	3/3	1.08± 0.81	3/3	0.79± 0.87	3/3
CK1	1± 0.23	3/3	0.76± 0.22	3/3	1.47± 1.84	3/3	11.98± 16.81	2/3
CK4	1± 0.22	3/3	0.31± 0.11	3/3	0.84± 0.48	3/3	1.53± 1.73	3/3
CK5	1± 0.49	3/3	0.1± 0.04	3/3	0.1± 0.05	3/3	0.27± 0.28	3/3
CK7	1± 0.6	3/3	0.58± 0.23	3/3	0.63± 0.28	3/3	3.07± 2.11	3/3
CK8	1± 0.35	3/3	0.75± 0.3	3/3	0.53± 0.3	3/3	0.92± 0.68	3/3
CK10	1± 0.16	3/3	0.29± 0.15	3/3	0.9± 1.03	3/3	1.63± 2.38	3/3
CK13tv1	1± 0.06	3/3	0.12± 0.09	3/3	0.22± 0.15	3/3	1.34± 0.93	3/3

	Control		INF		INF+Ibu		Ibu	
**Marker**	**mean ± SD**	**N**	**mean ± SD**	**N**	**mean ± SD**	**N**	**mean ± SD**	**N**
CK14	1± 0.13	3/3	0.73± 0.16	3/3	0.47± 0.12	3/3	1.53± 0.93	3/3
CK16	1± 0.09	3/3	0.23± 0.11	3/3	0.29± 0.17	3/3	1.65± 1.22	2/3
CK18	1± 0.23	3/3	1.03± 0.22	3/3	0.75± 0.32	3/3	0.79± 0.85	3/3
CK19	1± 0.22	3/3	1.18± 1.16	3/3	1.18± 0.93	3/3	1.53± 0.26	2/3
LOR	1± 0.46	3/3	0.49± 0.22	3/3	1.02± 0.85	2/3	2.69± 2.89	2/3
DSG3	1± 0.1	3/3	0.37± 0.16	3/3	0.47± 0.25	3/3	0.83± 0.85	3/3
AMY	1± 0.48	3/3	0.79± 0.17	3/3	0.46± 0.22	3/3	0.38± 0.43	2/3
CDH1	1± 0.32	3/3	1.13± 0.42	3/3	0.94± 0.55	3/3	0.93± 0.62	3/3
PECAM1	1± 0.28	3/3	0.82	1/3	0.47± 0.06	2/3	0.77	1/3
VEGF-A	1± 0.24	3/3	0.23± 0.04	3/3	0.24± 0.01	3/3	0.5± 0.27	3/3
VIM	1± 0.73	3/3	0.58± 0.42	3/3	0.91± 0.76	3/3	0.56± 0.52	3/3
VWF	1± 0.89	3/3	0.24± 0.28	3/3	0.24± 0.1	3/3	1.28	1/3
MUC1A	1± 0.62	3/3	3.43± 1.52 *Ibu	3/3	1.5± 0.78	3/3	0.53± 0.42	3/3
MUC1B	1± 0.6	3/3	6.73± 2.78 ***Control, INF+Ibu, Ibu	3/3	2.77± 1.69	3/3	0.81± 0.36	3/3
MUC3A	1± 0.98	3/3	2.71± 3.16	2/3	1.26± 1.06	2/3	2.34± 2.52	2/3
MUC5AC	1± 0.2	2/3	1.85	1/3	1.25	1/3	0.76± 0.17	2/3
MUC15tv2	1± 0.42	3/3	0.09± 0.08	2/3	0.09± 0.02	3/3	0.46± 0.21	2/3
MUC16	n/a	0/3	n/a	0/3	n/a	0/3	n/a	0/3
MUC18	1± 0.09	3/3	4.63± 2.68	3/3	1.81± 1.68	3/3	0.89± 0.56	3/3
MUC20	1± 0.54	3/3	0.82± 0.29	3/3	0.62± 0.35	3/3	1.43± 0.61	3/3
MUC21	1± 0.83	2/3	0.56± 0.23	3/3	0.67± 0.65	3/3	1.65± 0.86	2/3
AQP1	1± 0.2	3/3	0.14± 0.06	2/3	0.28	1/3	0.34± 0.19	2/3
AQP3	1± 0.26	3/3	0.37± 0.12	3/3	0.21± 0.05	3/3	0.76± 0.67	3/3
AQP5	1± 0.33	3/3	0.43± 0.07	3/3	0.21± 0.11	3/3	1.57± 1.16	3/3
AQP9	1± 0.72	3/3	9.94± 3.87 ***Control, INF+Ibu, Ibu	3/3	4.35± 3.41 **Control, Ibu	3/3	0.93± 0.04	2/3
AQP10	1± 0.07	3/3	0.56± 0.22	3/3	0.17± 0.12	3/3	1.38± 0.72	2/3
AQP11	1 ± 0.18	3/3	0.61 ± 0.19	3/3	0.34 ± 0.06	3/3	0.66 ± 0.07	2/3

Table S3. Overview of physicochemical properties of NSAIDs, accessed on public databases, and calculated permeability coefficients of NSAIDs in this study.

NSAID	XlogP3[1]	XlogD[2]	Permeability coefficient without inflammation and inhibitor
Celecoxib	3.4	4.01	0.44 ± 0.19 µm/min
Diclofenac	4.4	1.1	3.64 ± 0.84 µm/min
Ibuprofen	3.5	1.34	5.77 ± 1.48 µm/min
Piroxicam	3.1	- 1.52	4.86 ± 2.41 µm/min

[1] https://pubchem.ncbi.nlm.nih.gov (01.04.2024) [2] https://ebi.ac.uk (02.04.2024)

References

1. Lin GC, Leitgeb T, Vladetic A, Friedl H-P, Rhodes N, Rossi A, et al. Optimization of an oral mucosa in vitro model based on cell line TR146. Tissue barriers. 2020 Apr;8(2):1748459.

2. Mineta K, Yamamoto Y, Yamazaki Y, Tanaka H, Tada Y, Saito K, et al. Predicted expansion of the claudin multigene family. FEBS Lett. 2011 Feb;585(4):606–12.
